# Surgical Periodontal Management of Peripheral Ossifying Fibroma: A Series of Three Cases

**DOI:** 10.1155/2024/3683561

**Published:** 2024-03-11

**Authors:** Karo Parsegian, Roger M. Arce, Nikola Angelov

**Affiliations:** ^1^Division of Periodontics, Department of Diagnostic Sciences and Surgical Dentistry, School of Dental Medicine, University of Colorado Anschutz Medical Campus, Aurora, CO, USA; ^2^Department of Periodontics and Dental Hygiene, School of Dentistry, University of Texas Health Science Center, Houston, TX, USA

## Abstract

Peripheral ossifying fibroma (POF) is a benign swelling of the gingival connective tissue commonly associated with dental biofilm and biofilm-retentive dental appliances. In the present case report, we described three cases of POF with different clinical presentations and treatment approaches. The treatment consisted of the removal of supra- and subgingival calculus, followed by a flap surgery with excision of the entire lesion ensuring the inclusion of the periosteal bed. The first patient developed POF during her pregnancy that remained clinically noticeable postpartum. The second case represented a rare case of POF appearing on the palatal aspect of the anterior maxilla of an African American male. The third case represented POF that developed on the mandible, and contrary to the first two cases, it was excised using a diode laser and not a scalpel blade. All patients showed uneventful healing during follow-up appointments; however, poor patient compliance did not allow for evaluation of long-term healing responses and possible recurrence of the lesion. Within the limitations of this clinical report, it is evident that the periodontal surgical approach was effective in managing POF with stable short-term clinical outcomes.

## 1. Introduction

The 2022 World Health Organization classification categorizes odontogenic and maxillofacial bone tumors into (i) cysts of the jaw, (ii) benign odontogenic tumors, (iii) malignant odontogenic tumors, (iv) giant cell lesions and bone cysts, (v) bone and cartilage tumor-fibro-osseous tumors and dysplasias, (vi) bone and cartilage tumor-benign maxillofacial bone and cartilage tumors, and (vii) bone and cartilage tumor-malignant maxillofacial bone and cartilage tumors [1]. Peripheral ossifying fibroma (POF) is one of the most common types of fibro-osseous lesions with a reported prevalence of ~18-20% [2, 3] and the highest prevalence observed in the second and third decades [3–5]. Often confusing, POF is not a peripheral type of the central ossifying fibroma but rather a reactive gingival hyperplasia with signs of mineralization [6]. Clinically, the lesion represents a slowly expanding, exophytic, and well-demarcated gingival mass commonly originating from the interdental papilla [2, 3] and reaching ~1.2 cm in diameter on average [7]. POF is usually pedunculated [5] and pink to red [5] but can vary in subjects of different races and was ulcerated in up to ~66% of cases [4]. The lesion was more prevalent in the maxilla than in the mandible (56.2-61.5 vs. 38.5-43.8%, respectively) and in anterior sextants compared to posterior dentition [2, 3]. Clinical manifestations of POF commonly include gingival bleeding (40%) and pain (~3.3%), and up to ~6% of patients with POF display other signs and symptoms including poor oral hygiene [2]. Radiographically, POF develops without apparent involvement of the alveolar bone; however, superficial erosion of the bone can occur in some cases. A well-demarcated mass that contains scattered calcifications can be commonly observed on a computed tomography scan [8].

Dental biofilm and calculus accumulations, poorly finished or fitting dental appliances and restorations, and intraoral pathogenic microbiota are associated with up to ~41% of POF cases [7] suggesting their role as predisposing factors. However, the etiologic factor of POF remains to be identified. Some hypotheses suggest an osteogenic origin of POF due to the presence of mature bone and osteoid within the POF lesions [4, 5] and rare cases of POF on edentulous ridges not associated with the periodontal ligament (PDL) [9]. However, the odontogenic origin of POF from the PDL appears to be a predominant theory, which is supported by the localization of POF limited to the gingiva and near the adjacent permanent (and rarely, primary [7]) teeth. In addition, the PDL-associated origin of POF is consistent with histological features of the lesion represented by two distinct patterns: (i) a more frequent hypercellular pattern characterized by a dense fibrocellular proliferation, randomly dispersed focal deposits of calcified tissue of various shapes, architecture, and amounts, and (ii) dense fibrocellular to predominantly fibrous stroma with deposits of calcified osseous lamellae and trabeculae with circumferential osteoid [10]. Although cellular connective tissue is pathognomonic of POF, the type of calcifications can vary from osseous formations (woven, trabecular, and lamellar) surrounded by poorly mineralized osteoid, to cementum-like formations and dystrophic calcifications [4, 5].

The goal of the present case series study was to describe the periodontal management of three clinical cases of peripheral POF and discuss these findings in the context of the currently available evidence. The clinician-assessed outcomes included the excision of the lesion and the lack of its recurrent development. The patient-assessed outcomes included esthetic satisfaction and functional improvements (lack of adverse functional or esthetic effects). All procedures followed the ethical standards of the committee responsible for human experimentation (institutional and national) and principles of the World Medical Association Declaration of Helsinki 1964 (and its later amendments). The manuscript was prepared according to the 2013 CARE (Case Report) guidelines.

## 2. Case Presentation

In all three cases, the proposed treatment plan included (1) a hygienic phase with supra- and subgingival scaling; (2) a surgical phase with conservative gingival flap surgery, excisional biopsy, and gingivoplasty for esthetic recountering of the gingiva; and (3) a periodontal maintenance phase. Histological analysis was similar for all three cases, and the main features are summarized in Table 1.

### 2.1. Case #1

#### 2.1.1. Medical and Dental History

A Latin American female patient presented to the Advanced Periodontics clinic for comprehensive periodontal evaluation with a chief complaint of “swollen gums.” The patient reported the lesion first appeared about four months into her pregnancy when 18 years of age and persisted for three years postpartum. Pregnancy was reported as uneventful. The patient denied alcohol, tobacco, and recreational drug use. At the time of the baseline examination, the patient's medical, family, and psychosocial history was reviewed, and based on the classification of the American Society of Anesthesiologists (ASA), she was classified as ASA 1.

#### 2.1.2. Clinical Findings

**Figure 1:**
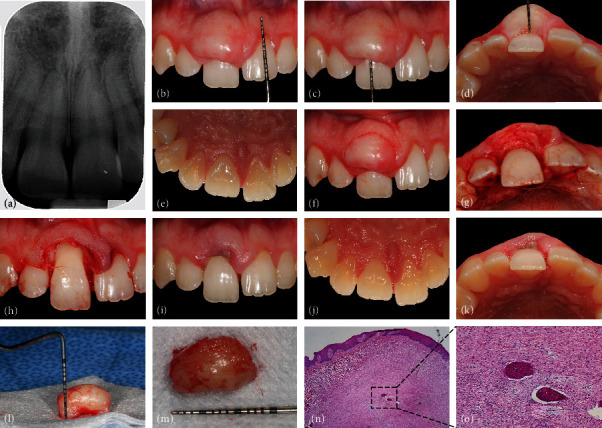
Clinical case #1. The lesion developed on a buccal aspect between teeth #8 and 9 with no apparent radiographic changes (a–e). The lesion was excised using a scalpel blade (f–h). Postoperative healing at 1 week revealed no complications (i–k). The patient did not present for further follow-up appointments. Tissue samples harvested during biopsy (l, m) were submitted for histological analysis to institutional oral pathology facilities, and histopathologic findings were found to be pathognomonic in the diagnosis of POF (n, o).

Figure 1(a) shows a periapical (PA) radiograph taken at the baseline appointment with radiographic signs of moderate (~15-30% of the root length) horizontal alveolar bone loss around tooth #8. Lamina dura appeared intact. Extraoral examination revealed no lymphadenopathy or other abnormalities. Oral cancer screening was negative. Oral hygiene was good to fair, evidenced by the lack of heavy dental biofilm and calculus deposits throughout the dentition. Figures 1(b)–1(d) show a round-shaped gingival mass on the buccal aspect of teeth #8 and 9 without extending into a palatal surface (Figure 1(e)). The lesion appeared similar in color to the adjacent gingiva, firm and sessile upon palpation, and regular in shape measuring 14 × 14 × 5 mm apicocoronally, mesiodistally, and faciolingually, respectively (in maximum dimension). The lesion was asymptomatic, and its surface demonstrated no clinical evidence of ulceration and bleeding. However, the clinical manifestation of the lesion was associated with the patient's esthetic concern and functional dysfunction since tooth #8 displayed Miller class 1 mobility and was shifted lingually out of alignment with other teeth in the maxillary dentition creating ~1 mm diastema between teeth #8 and 9. The periodontal evaluation demonstrated the presence of gingival inflammation evidenced by bleeding on probing (BOP), isolated clinical attachment loss (CAL) in the form of gingival recession, and probing depths (PDs) ranging from 1 to 3 mm.

#### 2.1.3. Diagnostic Assessment

Based on the 2017 classification of periodontal and peri-implant diseases and conditions [11], the patient was clinically diagnosed with non-biofilm-induced gingival disease on a reduced periodontium in the form of reactive processes. Provisional differential diagnoses of “3Ps” (pyogenic granuloma (PG), central giant cell granuloma (CGCG), and POF) and focal fibrous hyperplasia (FFH) were considered.

#### 2.1.4. Timeline and Patient Management

A one-minute preprocedure rinse with 0.12% chlorhexidine gluconate was performed. Supra- and subgingival scaling was performed around teeth #8 and 9 using ultrasonic and sharpened hand instruments (Gracey curettes) under local infiltration anesthesia with one carpule of 4% Septocaine (articaine +1 : 100,000 epinephrine). An inverse bevel, submarginal incision was performed using a #15C scalpel blade beginning at the mesial papilla of tooth #8 following the gingival contour and extending to the distal papilla (Figure 1(f)). The incision was made to the depth of the alveolar bone and designed to remove the lesion in its entirety. Upon lesion removal, a circumferential intrabony defect on the facial aspect of tooth #8 was clinically visualized (Figures 1(g) and 1(h)). The defect was measured ~4 × 4.5 mm buccolingually from the tooth to the facial alveolar bone and ~4.5 mm apicocoronally from the crest of the alveolar bone to the deepest part of the defect. The defect was fully debrided to ensure a complete removal of soft tissue. The patient was recommended to use a soft toothbrush to maintain adequate oral hygiene in the surgical area and over-the-counter analgesics of choice in case of discomfort.

#### 2.1.5. Outcomes

One week after the surgery, the gingival tissue in the surgical area appeared to heal uneventfully (Figures 1(i)–1(k)). Slight bleeding with minor to no signs of edema, erythema, and suppuration was observed. No other adverse or unexpected events were reported or observed. Home oral hygiene instructions were reinforced. The patient did not present for further follow-up appointments.

#### 2.1.6. Histological Evaluation

The excised lesion (~12.5 × 10 × 7.5 mm, Figures 1(l) and 1(m)) was placed in 10% neutral buffered formalin immediately after harvesting it during the surgery and submitted for histological analysis. The decalcified tissue was sectioned using a soft tissue microtome (3 *μ*m thickness) and stained with hematoxylin and eosin (Figures 1(n) and 1(o)). Histopathologic findings were pathognomonic in the diagnosis of POF.

### 2.2. Case #2

#### 2.2.1. Medical and Dental History

An African American male patient presented to the Advanced Periodontics clinic for comprehensive periodontal evaluation with a chief complaint of “a bump behind my teeth.” The patient reported that the lesion first appeared several months ago, but he was hesitant to consult a dentist. The patient denied alcohol, tobacco, and recreational drug use. At the time of the baseline examination, the patient's medical, family, and psychosocial history was consistent with ASA 1.

#### 2.2.2. Clinical Findings

**Figure 2:**
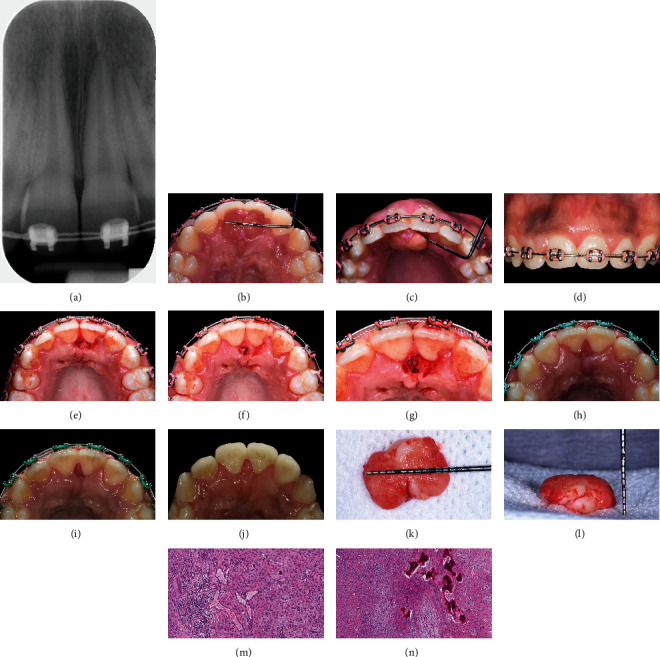
Clinical case #2. The lesion developed on a palatal aspect between teeth #8 and 9 with no apparent radiographic changes (a–d). The lesion was excised using a scalpel blade (e–g). Postoperative healing at 1-week (h), one-month (i), and three-month (j) follow-up appointments was found to be uneventful. Tissue samples harvested during biopsy (k, l) were submitted for histological analysis, and histopathologic findings were found to be pathognomonic in the diagnosis of POF (m, n).

Figure 2(a) shows a PA radiograph taken at the baseline appointment that revealed no radiographic signs of the alveolar bone loss, rather showed the alveolar bone crest to be located at the level of cemento-enamel junction (CEJ) suggesting a possible diagnosis of the excessive gingival display. Lamina dura appeared to be intact. Extraoral examination revealed no lymphadenopathy or other abnormalities. Oral cancer screening was negative. Oral hygiene was good to fair. Intraoral examination revealed a well-demarcated, round-shaped, pedunculated gingival mass on the palatal aspect of teeth #8 and 9 but not on the buccal aspect (Figures 2(b)–2(d)). The lesion appeared to originate from the interdental papilla. It was asymptomatic, nonulcerated, similar in color to the adjacent gingiva, firm and sessile upon palpation, and regular in shape measuring ~13 × 11 × 3 mm apicocoronally, mesiodistally, and faciolingually, respectively (in maximum dimension). No diastema was observed between teeth #8 and 9. The periodontal evaluation demonstrated a lack of CAL, PDs ranging from 1 to 3 mm, and the presence of gingival inflammation evidenced by BOP and gingival erythema.

#### 2.2.3. Diagnostic Assessment

Based on the 2017 classification of periodontal and peri-implant diseases and conditions [11], the patient was clinically diagnosed with non-biofilm-induced gingival disease on an intact periodontium in the form of reactive processes. Provisional differential diagnoses of “3Ps” (PG, CGCG, and POF) and FFH were considered. Since the patient was undergoing orthodontic therapy, consultation was performed with an orthodontist, who evaluated the patient and concluded that the lesions appeared to have no association with the mechanics of an orthodontic wire or trauma and were not related to the orthodontic tooth movement; mandibular incisors did not impinge the maxillary incisors.

#### 2.2.4. Timeline and Patient Management

A one-minute preprocedure rinse with 0.12% chlorhexidine gluconate was performed. Supra- and subgingival scaling was performed around teeth #8 and 9 using ultrasonic and sharpened hand (sickle scaler) instruments. The patient was administered local infiltration anesthesia using two carpules of 4% Septocaine (articaine +1 : 100,000 epinephrine). An inverse bevel, submarginal incision was made using #15 and 12 scalpel blades beginning at the mesial papilla of #8 following the gingival contour and extending to the distal papilla (Figures 2(e)–2(g)). The incision was made to the depth of the alveolar bone and was designed to remove the lesion in its entirety. Once the lesion was removed, a circumferential intrabony defect on the facial aspect of #8 was clinically visualized, and the soft tissue was removed until the defect was completely debrided. Sutures and postoperative management include prescriptions for 0.12% chlorhexidine gluconate mouthwash and 600 mg ibuprofen.

#### 2.2.5. Outcomes

One week after the surgery, the gingival tissue in the surgical area appeared to heal uneventfully as shown in Figure 2(h). Slight bleeding and moderate edema and erythema were observed. Sutures resorbed and were not observed in the mouth. Healing continued to be uneventful at one-month (Figure 2(i)) and three-month (Figure 2(j)) follow-up appointments. No adverse and unanticipated events were reported and observed at any time point. Home oral hygiene instructions were reinforced. The patient did not present for further follow-up appointments.

#### 2.2.6. Histological Evaluation

The excised lesion (~12 × 9 × 5 mm, Figures 2(k) and (l)) was placed in 10% neutral buffered formalin immediately after harvesting during the surgery and submitted for histological analysis. The decalcified tissue was sectioned using a soft tissue microtome (3 *μ*m thick sections) and stained with hematoxylin and eosin (Figures 2(m) and 2(n)). Histopathologic findings were pathognomonic in the diagnosis of POF.

### 2.3. Case #3

#### 2.3.1. Medical and Dental History

A Caucasian female patient presented to the predoctoral dental clinic for comprehensive periodontal evaluation with a chief complaint of “a bump in the mouth for a long time.” The patient reported that the lesion was first noticed about seven years ago. She had several consultations to have the lesion removed; however, no procedure was performed. Five years after the lesion was noted, the patient attempted to remove it herself by cutting it off using a pair of scissors; however, the lesion reappeared at a larger size within a few weeks. Medical history was significant for elevated blood pressure (126 × 80 mm Hg), class I obesity (BMI 32.6), and hormonal therapy for pregnancy. The patient denied tobacco, alcohol, and recreational drug use. At the time of the baseline examination, the patient's medical, family, and psychosocial history was consistent with ASA 2.

#### 2.3.2. Clinical Findings

**Figure 3:**
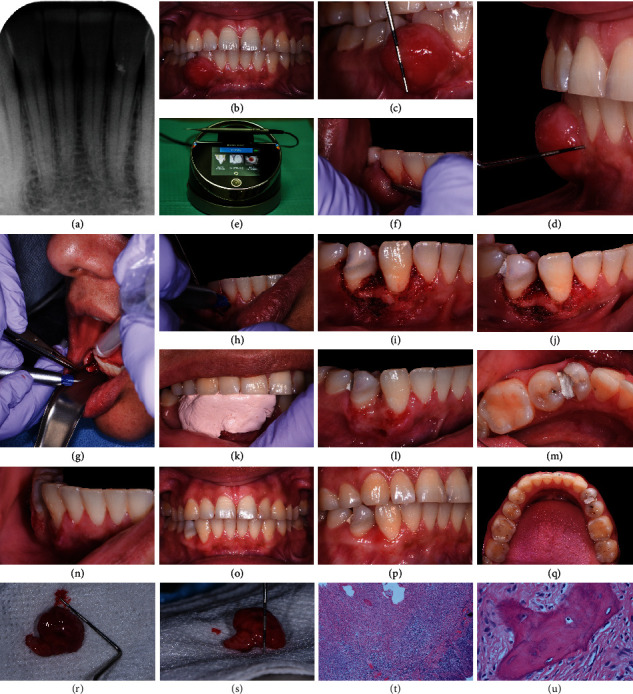
Clinical case #3. The lesion developed on a buccal aspect between teeth #27 and 28 with no apparent radiographic changes (a–d). The lesion was excised using the Biolase laser (940 nm wavelength) equipped with a 300 *μ*m tip and at an average power of 1 W (e–k). Postoperative healing at 1 week (l, m), 2 weeks (n), 4 weeks (o), and 6 weeks (p, q) revealed uneventful healing with no complications. The patient did not present for further follow-up appointments. Tissue samples harvested during biopsy (r, s) were submitted for histological analysis, and histopathologic findings were found to be pathognomonic in the diagnosis of POF (t, u).

Figure 3(a) shows a PA taken at the baseline appointment that revealed no radiographic signs of alveolar bone loss. Lamina dura appeared to be intact. Extraoral examination revealed no lymphadenopathy or other abnormalities. Oral cancer screening was negative. Oral hygiene was poor with moderate to heavy dental biofilm accumulation throughout the dentition (the original O'Leary plaque score of 62%) and BOP of 38%. The patient had a high caries risk. Further intraoral examination revealed a well-demarcated, round-shaped, pedunculated gingival mass on the buccal aspect of teeth #27 and 28 (Figures 3(b)–3(d)). The lesion was asymptomatic, nonulcerated, erythematous, firm, and sessile upon palpation and regular in shape measuring ~12 × 10 × 7 mm apicocoronally, mesiodistally, and faciolingually, respectively (in maximum dimension). Teeth #27 and 28 had no clinically detectable mobility. The periodontal evaluation demonstrated isolated CAL in the form of gingival recession, PDs ranging from 1 to 4 mm, and gingival inflammation evidenced by the presence of BOP and gingival erythema.

#### 2.3.3. Diagnostic Assessment

Based on the 2017 classification of periodontal and peri-implant diseases and conditions [11], the patient was clinically diagnosed with biofilm-induced gingivitis without local contributory factors. In addition, a diagnosis of non-biofilm-induced gingival disease (in the form of reactive processes) was made. Provisional differential diagnoses of “3Ps” (PG, CGCG, and POF), FFH, peripheral giant cell granuloma, and peripheral fibroma were considered.

#### 2.3.4. Timeline and Patient Management

A one-minute preprocedure rinse with 0.12% chlorhexidine gluconate was performed. The patient was anesthetized using one carpule of 2% lidocaine +1 : 100,000 epinephrine. Supra- and subgingival scaling was performed around teeth associated with the lesion, using ultrasonic and sharpened hand instruments. The lesion was then retracted away from the teeth using a periosteal elevator and excised using a Biolase laser (940 nm wavelength) equipped with a 300 *μ*m tip and at an average power of 1 W (Figures 3(e)–3(i)). A part of the buccal plate was exposed between teeth #27 and 28. Since tooth #28 had a fractured crown with no pulp exposure, zinc oxide-eugenol cement was placed temporarily to protect the tooth during and after the surgery (Figure 3(j)). The defect was measured ~4 × 4.5 mm buccolingually from the tooth to the facial alveolar bone and ~4.5 mm apicocoronally from the crest of the alveolar bone to the deepest part of the defect. Hemostasis was achieved. Coe-Pak™ surgical dressing was placed to protect the surgical area (Figure 3(k)). Postoperative management included a prescription for 0.12% chlorhexidine gluconate mouthwash.

#### 2.3.5. Outcomes

One week after the surgery, Coe-Pak™ was removed, teeth were scaled and polished, and the gingival tissue in the surgical area appeared to heal uneventfully (Figures 3(l) and 3(m)). Moderate edema and erythema with no signs of bleeding and suppuration were observed at 2 weeks (Figure 3(n)), 4 weeks (Figure 3(o)), and 6 weeks (Figures 3(p) and 3(q)). The patient did not present for further follow-up appointments.

#### 2.3.6. Histological Evaluation

The excised lesion (~8 × 11 × 4 mm, Figures 3(r) and 3(s)) was placed in 10% neutral buffered formalin immediately after harvesting and submitted for histological analysis. The decalcified tissue was sectioned using a soft tissue microtome (3 *μ*m thick sections) and stained with hematoxylin and eosin (Figures 3(t) and 3(u)). Histopathologic findings were pathognomonic in the diagnosis of POF.

## 3. Discussion

In the present study, we described three cases of POF in patients with various medical statuses, the lesion location, and the mode of performing the lesion excision. Since POF is a hyperplastic reactive lesion of a non-neoplastic nature [3], all reported cases were treated by conservative and not radical surgical excision [12]. This is consistent with previously reported periodontal surgical approaches to manage POF cases such as coronally positioned and laterally positioned flaps [12]. Since POF commonly occurs in the anterior maxillary sextants, the patient's esthetic demands can require advanced soft tissue management, such as subepithelial connective tissue grafting [13]. Some authors also used collagen matrix mucografts to augment the amount of connective tissue in the surgical area [14]. Clinical presentation may play a role in how soon the lesion is excised (~6 vs. 24 months for ulcerated and nonulcerated POF lesions, respectively) [4].

Only a limited number of studies reported the prevalence of POF during pregnancy and postpartum, as shown in our case #1. An older study that included thirty-two “pregnancy tumors” has shown that 28.1% of them represented POF, and one case was recurrent [15]. Another older study reported a case of POF in the mandibular anterior and premolar sextants that was diagnosed in the sixth month of pregnancy [16]. A more recent study reported a single case of POF in a 25-year-old female who developed POF during the second trimester of her pregnancy, but the surgical excision was performed postpartum [11]. The mechanisms contributing to the development of POF during pregnancy include low-grade gingival inflammation associated with dental biofilm and calculus accumulation as well as hormonal changes (increased levels of estrogen and progesterone leading to increased vascular proliferation) [15]. Another interesting observation in our case #1 was the proposed origin of the lesion from the straight buccal aspect of the tooth and not from the interdental papilla, as it was commonly reported for POF.

The reported prevalence of POF was markedly higher in females compared to males (up to 6 : 1, respectively [17]) and in Caucasians (69-79%) compared to other races [4]. In the present study, case #2 was a rare case of POF developing in an African American male.

Published periodontal surgical approaches commonly used the scalpel blade to excise POF. However, the use of the laser has become increasingly popular in recent years. A neodymium-doped yttrium aluminum garnet (300 *μ*m surgical fiber optic tip, 4 W power, no wavelength was reported) was used to excise POF lesions in two adult patients [18]. The diode laser (1 W power, 940 nm wavelength) was used to excise a POF lesion in a three-month-old infant [19]. Another study reported on the use of the Biolase diode laser (400 *μ*m surgical fiber optic tip, 1.2 W power, and 940 nm wavelength) to excise a POF lesion in an adult patient [20]. In these studies, immediate and effective hemostasis was achieved with uneventful healing. These findings correlate with our clinical observations in case #3, where the use of the diode laser at 1 W power and 940 nm wavelength to excise the POF lesion promoted hemostasis and led to uneventful healing.

Although all patients were offered complimentary adult prophylaxis, they did not return for long-term follow-up appointments despite several phone call reminders, which is a common limitation of all three cases. Therefore, the patient's perspective on the received treatment remains unclear. The reported rate of recurrence for POF was higher than expected for a benign reactive proliferation (up to ~66% of cases) and forming the lesion with the same histological signs as the original one [4]. However, it is important to note that the recurrence rate varied substantially in different studies and could be as low as 5% [7]. Cases of multiple POF recurrences in the same patient were also described [7] that could be due to incomplete surgical excision of the lesion, repeated injury to the surgical area, and/or inability to eliminate contributing irritants. These data further highlight the importance of complete debridement of the lesion during periodontal surgical therapy. At the same time, it raises an interesting hypothesis that the timing and rates of POF recurrence excised using laser can be lower compared to the lesions excised by the blade, which is a focus of our future studies.

## 4. Conclusion

Within the limitations, the present study demonstrated successful short-term management of POF lesions using a simple gingival flap approach. Based on the literature evidence and standards of periodontal care, regular and timely follow-up appointments are essential to monitor the recurrence of POF lesions to ensure their complete elimination. Patient awareness of the recurrent nature of POF lesions should be emphasized. Both scalpel and diode laser approaches used in our cases appear to be equally effective surgical tools; however, further studies are needed to compare the recurrence rates of POF excised using these approaches. Regardless of the surgical tools and techniques used to manage cases of POF, patients need to be educated on the importance of regular and long-term postsurgical follow-ups.

## Figures and Tables

**Table 1:** Histological analysis. Common main histological features of all three cases of POF are listed.

Histological analysis
(i) Mucosal mass surfaced by parakeratinized stratified squamous epithelium (ii) Tissue proliferation constituted a nodular configuration, devoid of surface epithelium, also covered by a thick fibrinous layer (iii) Mesenchymal spindle cell proliferation (iv) Intertwining bundles of collagen supporting numerous haphazardly arranges of mesenchymal, fibroblast-like cells interspersed by occasional small blood vessels engorged with erythrocytes (v) Focal areas of osteoid and new bone formation (vi) Ulcerated benign cellular mesenchymal tissue proliferation supporting bone and cementum-like calcified deposits (vii) Numerous bone trabeculae containing osteocytes with lacunar and basophilic cementum-like calcified deposits (viii) Chronic inflammation of the underlying lamina propria (ix) Neutrophils and extravasated erythrocytes and occasional basophilic bacterial colonies (x) Inflammatory cells mostly included neutrophils and lymphocytes (xi) No evidence of malignancy

## Data Availability

The data supporting this study's findings are available from the corresponding author (KP) upon reasonable request.
